# How to mobilise users' experiential knowledge in the evaluation of advanced technologies and practices in Quebec? The example of the permanent users' and relatives' panel

**DOI:** 10.1111/hex.13964

**Published:** 2024-01-05

**Authors:** Marie‐Pascale Pomey, Sandra Pelaez, Enora Le Roux, Oliver Demers‐Payette, Marie‐Claude Sirois, Louis Lochhead, Isabelle Ganache, Louise Normandin, Audrey L'Espérance, Michèle de Guise

**Affiliations:** ^1^ Research Centre of the University of Montreal Hospital Centre Montréal Québec Canada; ^2^ Centre d'excellence sur le partenariat avec les patients et le public Montréal Québec Canada; ^3^ Department of Health Policy, Management and Evaluation, School of Public Health University of Montréal Montréal Québec Canada; ^4^ Institut national d'excellence en santé et services sociaux (INESSS) Montréal Québec Canada

**Keywords:** evaluation, health technology assessment agency, social services, users' experiences, users' panel, users' perspective

## Abstract

**Introduction:**

With the purpose of supporting scientific professionals and helping them to better integrate the expertise of users in their work, a users' and relatives' panel (URP) was set up at the National Institute for Excellence in Health and Social Services in Quebec (INESSS), Canada for the social services and mental health directorate. URPs are advisory structures that mobilise the experiential knowledge of people affected by various issues.

**Objectives:**

The objective of this study is to assess from a diverse stakeholders' perceptions: (1) the experience of developing and implementing the URP within the context of an Agencies for Health Technology Assessment and Assessment of Social Services (AHTAASS), (2) the contribution of such a URP, (3) the challenges encountered and (4) the perspectives of improvement for the following years.

**Methodology:**

We conducted a qualitative descriptive evaluation study. Nineteen interviews were conducted: six with URP members and 13 with staff representatives. The documents related to the creation of the panel, the URP minutes summarising the discussions and the reports published during that period were collected and analysed. Following a preliminary round of data analysis, a debriefing meeting was conducted with a few participants to validate the results.

**Results:**

The panel was set up as part of the INESSS' desire to better integrate experiential knowledge into its recommendations. Twelve projects were presented to the panel on various themes. The URP enabled health professionals to consider dimensions they had not identified, to better integrate the experiential data collected from users into their work and to develop recommendations that made more sense to users. Panel members and INESSS professionals learned to work together, moving the working methods from consultation to collaboration and even coconstruction. Based on the panel's significant contribution, the INESSS decided to maintain it and to strengthen its place in its system to better integrate the experiential knowledge of users into its work.

**Conclusion:**

This research illustrates how AHTAASS can set up a URP composed exclusively of users, and how it can contribute and be evaluated. It shows that URPs are structures that value the sharing of experiential knowledge of its members, humanise decision‐making and give meaning to the work done by scientific professionals.

**Patient or Public Contribution:**

One patient–researcher has contributed to the preparation and writing of this manuscript.

## 1 INTRODUCTION

Agencies for Health Technology Assessment and Assessment of Social Services (AHTAASS) are increasingly called upon to integrate the experiential knowledge of patients, users, relatives and citizens into their work.^1^ In this context, Quebec's agency, the Institut national d'excellence en santé et services sociaux (INESSS),^2^ is no exception,^3^ especially as in Québec, in 2018, a framework for the partnership approach between users, their families and health and social service provider^4^ encourages all healthcare organisations to develop initiatives that mobilise patients' experiential knowledge. In this context, INESSS is developing a policy that integrates the points of view of citizens, patients and caregivers^5^, ^6^ and has set up an Office of Methodologies and ethics to support the participation of citizens, users, patients and their families in its various activities. This includes a patient coordinator and specialists in stakeholder engagement methodologies to support cultural change and develop innovative projects, whether in the field of drugs, technologies or interventions in healthcare^5^, ^6^ and social services. In the field of social services, INESSS's Direction de l'évaluation et du soutien à l'amélioration d'interventions—services sociaux et santé mentale (evaluation and support for the improvement of interventions—social services and mental health) (DESA), has decided to give greater recognition to experiential knowledge in its evaluation work, by creating, in 2019, a permanent users' and relatives' panel (URP) to contribute, on a regular basis, to the production processes of this work. This initiative was not embedded into a specific national or provincial policy.

Users' panels are consultative structures that mobilise the experiential knowledge of people affected by various issues. These panels are frequently observed in healthcare establishments, particularly in mental health.^7^, ^8^, ^9^ They enable regular consultation with people who are affected by the targeted issues to benefit from their experiential knowledge and thus ensure that their point of view is taken into consideration.

At the INESSS, the URP is not systematically requested for projects, and the decision to present the file to the URP rests with the project leader. The URP's mandates involve: (1) providing input to teams of professionals at various key stages of scientific production (project scoping, composition of working committees, consultations with users and their families, drafting of findings and recommendations, etc.); (2) enlightening scientific professionals on emerging concerns; (3) alerting them to issues linked to the acceptability and applicability of recommendations; (4) supporting initiatives to involve experts from the field in production processes; (5) contributing to knowledge transfer strategies that involve users and their families. The URP is called upon to give its opinion at every stage of the evaluation process, from scoping to the release of findings and recommendations. It is thus consulted on a wide variety of themes, methodologies and issues raised by the evaluation.

The objective of the present study is to assess: (1) the experience of developing and implementing the URP within health and social services technology and intervention assessment agencies; (2) the contribution of such a panel by focusing on diverse stakeholders' perceptions of their experiences; (3) the challenges encountered; and (4) the prospects of improvement for the following years.

This research aims to reach all AHTAASSs who are developing strategies for involving users in their work or those who wish to do so, as well as people in healthcare establishments who are interested in consulting users to help them make decisions most in line with their experienced needs. Thanks to INESSS's experience, we can provide a number of recommendations to guide them.

This study received ethical approval from the Research Ethics Committee (2020‐8803, 19.350) of the Centre de recherche du Centre hospitalier de l'Université de Montréal.

## 2 METHODOLOGY

### 2.1 Study design

We conducted a qualitative descriptive evaluation study, an eclectic design frequently used in healthcare when: (a) the particular phenomenon to be explored is in a natural state and remains unexplored; (b) the sample size is limited; (c) the data is sought to develop and/or refine an intervention; and (d) low theoretical interpretation and flexibility of methods are sought.^10^, ^11^, ^12^, ^13^

### 2.2 Participants, data collection and procedures

At the time the study was conducted, the URP was already running. To enhance the understanding of the context in which the panel was inserted, sampled participants were selected following a proximal‐to‐distal logic regarding both involvement (i.e., role played) and engagement (i.e., commitment towards the development and sustainability of the panel). As such, all panel members were interviewed first, followed by scientific professionals—from those closely working with them to those leading various institutional units, and finally with the institutional vice‐president. The topics that were discussed during the interviews were related to: (1) the process of setting up the URP; (2) the perception of the URP's contribution; (3) the challenges faced; and (4) the perspectives and prospects for improvement in the following years (see Appendix S1).

In addition, documents related to the creation of the URP, minutes summarising the panel discussions and the report published during that period were collected.

All participants were members of the institution; thus, they were informed of the purpose of the study and contacted through institutional venues. They all filled out a consent form to be part of the research project in which they agreed to have the discussion recorded. The interviews conducted on teams were, therefore, all recorded and transcribed.

To quote verbatims and to prevent participant identification due to the small‐size institution, all participants were assigned an identification code: PM for panel members and IM for institutional members.

### 2.3 Data analysis

The following steps were observed in the present study to analyse the qualitative data^13^: (a) in‐depth reading of the first four transcriptions and identification of key ideas; (b) development of a preliminary analytic framework with four themes (initiation, implementation, lessons learned and improvement) and 15 subcategorises (15) based on both Rogers'^14^ and Nancarrow's et al.,^15^ as well as in the nature of the information discussed in the interviews; and (c) constant comparison of codes that led to the development of the final coding scheme. The two researchers (M. P. P. and S. P.) codeveloped the codebook, conducted the thematic analysis independently in N‐Vivo edition 11, and discussed together which verbatims illustrated the best themes to be integrated into the manuscript. Notably, the preliminary results were submitted to the URP and the INESSS' team members during a 2‐h validation meeting in December 2022. Their comments were integrated in the final results.

In addition, all the documents collected were analysed on one hand to reconstruct the timeline of the creation of the panel and on the other hand to study how URP members are identified in the INESSS products.

## 3 RESULTS

### 3.1 General results

The 19 people approached to participate in the study were all interviewed individually, between March 2021 and February 2022, that is, the five panel members involved since its creation; eight people from DESA (scientific professionals [*n* = 3], coordinators [*n* = 3], managers [n = 2]); five people from the other two directorates (Directorate for the Evaluation of Drugs and Technologies for Reimbursement Purposes [*n* = 1], Directorate for the Evaluation and Relevance of Healthcare Intervention Methods [*n* = 1]) and the Ethics and Partnership Methodology Office (*n* = 3). Interviews lasted an average of 49 min (between 26 and 84 min—median 52 min). In all, the panel was consulted on 12 files during this period (see Appendices S2 and S3).

### 3.2 Process of setting up the panel

#### 3.2.1 Initial concept

The idea of creating the URP in the DESA originated from an activity organised by the INESSS, which focused on the ways in which citizens, users, patients and their families are involved in health and social services technology and intervention assessment agencies:

It was at an event organized by the INESSS, which I attended, that I learned that a hospital in Montreal had established a mental health user panel. I immediately found the idea very interesting. I thought it was excellent that people receiving care, or their loved ones could be consulted and share their experience to improve the services given to users. That's when I said to myself, why not do this to improve our work? (IM)

To establish this, various options emerged and were analysed:

I didn't want to come up with a mandate proposal, with objectives, with a purpose. I also didn't want to determine whether we should go for thematic groups (mental health, addiction, homelessness, troubled youth, physical disability, intellectual disability, autism spectrum disorder, seniors losing their autonomy, etc.) consulted on an ad hoc basis, or whether we should start with an interdisciplinary committee. So, we consulted the office in charge of user engagement methodologies and called on the Centre of Excellence on Partnership with Patients and the Public (CEPPP) to support us in the Panel's reflection. In the end, we decided to go for a multi‐clientele panel. (IM)

#### 3.2.2 Recruiting and training members

URP members were recruited on the basis of four pre‐established selection criteria, based on good practice and adjusted to the context of social services^16^: (1) to have significant experience of using social services, (2) to have taken a step back from their personal situation with the services, (3) to express themselves clearly, are not shy about saying things, and are critical thinkers and (4) to have a constructive and respectful attitude. These qualities were tested through interviews and role‐playing exercises led by CEPPP.

Recruitment involved two strategies. The first was to establish a collaboration with the CEPPP^17^ a centre that develops best practices in the field of partnership. CEPPP issued a call for applications to its network of user–partners, which includes around a 100 people. The second call went out to DESA employees. A total of 10 people expressed interest; seven were interviewed by the patient coordinator and the DESA director, and five were selected with varied profiles in addition to the INESSS patient coordinator, for a total of six members including the patient coordinator (see Table 1). Particular attention to diversity in perspectives (user, caregiver, parent, youth), coverage of issues/conditions addressed by the DESA team and previous experiences of engagement, which helped with the strategic and methodological role the panel would play, has been applied for the recruitment. Following their selection, URP members received an 8‐h training course, delivered by two CEPPP members. No recruitment happened before the training to align as much as possible patients' experience and knowledge with the aims of the panel. This training took place in person at the INESSS and covered the operating procedures of INESSS, DESA and the future panel, as well as panel expectations and requirements.

To set up the panel, management benefited from the support not only of CEPPP for the training [of panel members and DESA professionals] but also of the Ethics and Partnership Methodology Office. We started brainstorming with the patient coordinator and another expert from the office. They helped us with the theoretical aspects of recruitment and selection criteria, and with the practical aspects of running the panel. (IM)

**Table 1:** URP members' profile.

Sex	Age	Experience	Areas covered
Male	Between 60 and 70 years	Caregiver of a person with a disability	Physical impairment
Female	Between 60 and 70 years	Caregiver of an elderly person	Elderly people losing their autonomy
Male	Between 20 and 30 years	Mental health conditions	Mental health
Female	Between 40 and 50 years	Mother of a child with a disability	Intellectual disability
Male	Between 50 and 60 years	Father of a child with a disability	Physical and mental disability
Female	Between 20 and 30 years	Mental health conditions	Mental health, addiction, homelessness, troubled youth

Abbreviation: URP, users' and relatives' panel.

The selection and preparation process were well‐received by URP members.

The whole process leading up to my selection was very professional. I also really appreciated the training we received. It gave me a better understanding of what I could contribute to the INESSS's work. I also appreciated the first year, when we gradually got a better idea of our role. (PM)

#### 3.2.3 Operating methods

The term of office of members was not determined at the time of its creation. URP meetings take place 4–5 times a year. Panel members receive documents pertaining to the upcoming meeting in advance, so that they may prepare for the discussion. The meetings are led by the patient coordinator and DESA management.

The panel took a year to establish the foundations of its operation:

When we recruited the members, there was a ‘pre‐panel’ period to make sure we had a reflective space together. It took a good year, we had meetings, and we had CEPPP's support. We received training from CEPPP, for both management and panel members. We got used to it, and it was an extremely rich experience. At first, I wasn't convinced that we were multi‐clientele, but the panel members said to me: listen, in the end, we're all individuals with highly complex situations. We feel comfortable and interested, to be able to participate with our experiential experience on a more general level, and to have the concerns of users and loved ones that can reach all clienteles. (IM)

Before the pandemic began, meetings were face‐to‐face at the INESSS offices and lasted 1 day and lunch was provided. Beginning in March 2020, all meetings were held on TEAMS and were conducted over a half day. In addition, compensation of $CA 35/hour and for travel expenses is given to the URP members. To ensure optimal functioning of URP meetings, URP members have signed a written confidentiality contract. Additionally, they also approve an audio recording of the meeting to facilitate the production of meeting reports.

At each meeting, between three and four projects are presented by research agency professionals who ask pointed questions to URP members to respond to their needs. At these meetings, the project is presented by a scientific professional, then the members are invited to comment or suggest areas for improvement. The panellists' contributions vary according to the time and nature of the project presented. They may intervene on evaluation issues that have not yet been identified (concept note); on how to collect users' points of view (carrying out the mandate), improving the interpretation of results (carrying out the mandate), or reacting to knowledge transfer tools that have been developed. Suggestions are then incorporated into the fulfilment of the mandate. Between 2019 and May 2022, 12 projects were submitted to the URP in various fields (see Appendix S1).

### 3.3 Perception of URP added value

#### 3.3.1 Perceptions of URP members

Panel members provided examples of their various contributions. They could address the question of how to conduct a field survey…

For example, when we were asked to develop an interview grid for people living in long‐term care facilities (CHSLDs) or residences. (…) I made suggestions on how we could formulate the questions, and then I tried to give little tips, like in the discussion at the start, we could explain what hearing health is before asking questions with terms that aren't concrete or that don't make sense. (PM)

… Or about the impact that a tool like a questionnaire can have in gathering information about a situation:

One day we were told: ‘Here's the grid we could use to meet people’, and my alarm system went off, saying ‘Careful, what are you doing? You have to be careful with the tools’. I remember very well that I came back to a theme that's dear to me: a tool is all very well, but it can also be an *instrument of mass destruction*, depending on the person who's going to use it and the spirit that drives it. (PM)

They also pointed out that they enabled teams to identify areas that were more sensitive and required more attention than originally planned:

Everything to do with specific learning disorders [in children] was indeed something that was less well documented, because of the difficulties in linking the health network and the school environment. During the panel discussion, I insisted on this dimension and asked for additional empirical data to be collected. I also asked for additional analyses of the state of practice, to better understand why some things work well and others don't. I think I and my other colleagues were able to bring some nuance to the interpretation of the data. (PM)

I think we've helped the scientists at INESSS acquire new reflexes. Initially, when they quoted the people they had met, they quoted them in this order: managers, clinical experts, and parents … Why not put parents first? They're the first to experience the problems described in the state of practice or in the documents. So why not put them first? I think they've understood, and they've started to change the way they present their results. (PM)

However, panel members highlighted that it wasn't always easy to see the contribution they had made overall to a given mandate.: ‘I guess we were useful, but we don't get much feedback during the meetings. But we are the recipients of the reports, so we can see our contribution in action when reading the reports’ (PM).

#### 3.3.2 The perception of INESSS' members

From the research agency professionals' point of view, the panel adds depth to their mandate by giving them a deeper understanding of the subject at hand:

It's a permanent body (…) that evolves in its influence, to ensure that INESSS better integrates the perspective of those affected … The panel's added value is to help the stars align themselves on a project‐by‐project basis, so that the voice of those affected is taken into account and contributes to the project… (IM)

They are able to anticipate or raise issues that research agency professionals can't see:

They are a great help, (…), they will alert us to certain items. They act preventively: ‘Watch out for this’ or ‘Have you thought about this or that’? During [the completion] of projects, if we get (…) contradictory results, they'll bring in their interpretations that we weren't expecting. We can challenge them with this (…) In fact, research agency professionals are often surprised to see the extent to which they take us to another level of understanding of our problem. In my opinion, they are (…) really indispensable (…): both upstream and during the project. (IM)

They help determine the best strategies for gathering the views of the populations affected by the project:

For the perinatal and early childhood intervention project (SIPPE), panel members were called in at the outset because we wanted to hold consultations with users. We looked at the interview grid with them, and the type of questions we could ask them, knowing that SIPPE users are populations with very specific characteristics. At first, the grid was very long, but they helped us to reduce it. They pointed out that some questions didn't specifically reflect the needs of families. We presented their proposals to the committee of experts who were assisting us with the mandate, and they found it highly relevant too. (IM)

They help to contextualise data from the literature, which most often comes from cultural backgrounds different from Quebec's: ‘The fields of knowledge we investigate are sometimes disconnected from our Quebec reality. Discussing them with URP members enables us to better transpose the results of these studies to our context’.

They were also asked to help distribute the recommendations:

They give us ideas on how to better distribute our recommendations. They make us aware of knowledge transfer tools. It's a learning process for us, but along the way, they've also learned to take us there, to dare to do it, and to come up with more and more good ideas. And the fact that they've become acclimatized to our reality and our needs means they know how they can contribute better. Increasingly, we're working together, not so much separately, and finally we say to ourselves, ‘I don't like soup so much, I'd rather have cake!’ So why don't we make a cake? (IM)

Ultimately, they contribute to the scientific rigour of the work carried out by INESSS:

To have people who, on a permanent basis, work with management, work with the INESSS, with the concern to contribute from the inside at the scientific level and in a rigorous way to the problems they experience, is the main contribution for me.

#### 3.3.3 The perception of the common learnings achieved

Over and above the panel's contribution, the experience of interaction between URP and INESSS members has led to different awareness and interactions.

On the research agency professionals' behalf, it highlighted the fact that it's not easy to present a case in front of the panel:

The first time I came to present before the panel, I was very anxious. I didn't really know what to expect, and I didn't feel I'd worked hard enough on my presentation. I had the impression that I hadn't mastered my file as well as I thought. I was afraid I wouldn't be able to answer the questions. It took me several meetings with the panel to gradually become less afraid of being judged. (IM)

For panel members, the process can be just as confronting. As the members are not experts in all the subjects covered, they too feel the imposter syndrome. But over time, they have learned to trust the process and recognise the overall URP expertise:

At the beginning of my participation in the panel, I didn't feel comfortable intervening on subjects I hadn't mastered. But I gradually realized that my experience in other fields could be of interest to INESSS, and that I shouldn't shy away from asking questions and making connections with other situations. This really helped me to assume my role even more and to make the most of the meetings. (PM)

I sometimes feel bad about intervening in a problem I don't master. I feel like an impostor. It's not always easy to live with. (PM)

Finally, by confronting these two insecurities, the research agency professionals developed empathy for the panel members:

It's good to hear from panel members. They've always impressed me with the depth of their questions. I realized that I didn't have a detailed knowledge of the issues I was dealing with, as I hadn't experienced these situations first‐hand. This sometimes caused me great difficulty. I would never have imagined that it could be so distressing for them too. (IM)

### 3.4 Challenges faced

As far as membership is concerned, panel members highlighted the lack of cultural diversity among members, as well as the possibility of recruiting other profiles:

We could have more cultural diversity. Because at the moment, our committee is very, very white… (laughs) […]. We could have more varied profiles, such as someone on the autism spectrum. Age‐wise, I think it's great, we have people of all ages … except children. (PM)

From the point of view of agency professionals, the process for using the URP is not precise. There are no explicit criteria for assessing whether a project should be submitted to the URP, nor is there a consensus on the most appropriate time to submit it in the project implementation process, which may lead some agency professionals to hesitate to resort to the URP.

Right from the start of a project, I think we can call on the panel right from the pre‐framing and framing phase. I think it could be an interesting way of taking off some of the blinders we're wearing and looking at the bigger picture. Then, at the end, to see and discuss how it will be received by the population. Will there be any questions? A bit like we do with professional orders. (IM)

I'm not opposed to presenting it, it's just that I find it confusing as to what we want to get out of it. (IM)

Another issue raised by agency professionals is how to highlight the contribution of Panel members in INESSS reports. Their contribution is most often mentioned in the methodology and validation process, as well as in the results section, when their words are reported. However, there are no guidelines on how to proceed. In addition, URP members raised the point that they would appreciate feedback from agency professionals on the added value of their contribution to producing the reports.

Finally, the interviews, conducted with members of departments other than DESA, raised awareness of the panel's low profile.

I learned about the User Panel very recently at an in‐house presentation. I'd never heard of the initiative, so I thought it was really interesting. Then, after that, we got into the pandemic wave and, to be honest, it didn't really come up again. So, I don't know much about what this panel does. (IM)

I know it's been around for 2 years; I know it's in the social services field, but the activities, the contributions, I've never heard of it. (IM)

## 4 PERSPECTIVES AND AVENUES OF IMPROVEMENT

As far as prospects are concerned, DESA sees real added value in the URP for its work. It has decided to ensure its continuity by improving the way it operates and considering new developments.

The first proposal was to recruit new members to bring in additional expertise and people from other cultural backgrounds.

We started with 6 members, to test our model. We're now ready to integrate new members to ensure that we have all the expertise we need for our work around the table, as well as opening up to more cultural diversity. (IM)

It was also agreed that the URP should be consulted on all new mandates entrusted to DESA, to ensure that the various issues to be covered are discussed. Such an exchange would help build a common vision of the issues between the scientific agency professionals in charge of the mandate and the panel members.

We have a common need to go beyond a formal framework of discussion around an implementation plan that we will all devise on our own. We need to create a discussion space where we can easily approach a new mandate with Panel members, so that they alert us as early as possible in the process to issues we might not see. (IM)

I like this proposal, it would allow us to move away from a very scientific framework to talk emotionally about our perceptions of the issues based on our lived experience, with less pressure to respond to a well‐defined order. (PM)

A more in‐depth examination of how to better highlight the contribution of URP members in INESSS publications was proposed.

In terms of knowledge transfer within DESA, it was proposed to present experience feedback cohosted by scientific agency professionals and panel members.

Finally, to make the URP better known within the INESSS, it was suggested to: (1) put an item on the agenda of the Management Committee at least three times a year on the activities carried out by the panel; (2) enable other departments to call on the panel if relevant; (3) produce scientific webinars accessible to all on the panel's activities, cohosted by DESA scientists and panel members; (4) present the results of this evaluation to all INESSS members.

## 5 DISCUSSION

### 5.1 Operating procedures

The URP was set up to promote the involvement of users at a strategic level within an AHTAASS, to take greater account of the experiential knowledge of people affected by a range of social problems, including physical, intellectual and other forms of rehabilitation, as well as mental health. This practice is not widespread in these agencies.^18^ For example, the Canadian Agency for Drugs and Technologies in Health (CADTH) consults patients, families and communities for its various projects but has not yet created such a panel.^19^ Several facilitating factors stood out in its creation, such as (1) the commitment of DESA management to coconstruct a vision of the URP's role with its members, (2) the mobilisation of methodological expertise from within and outside the INESSS, (3) the allocation of financial and human resources to make it wok and (4) the cohosting of the panel by a user and a member of management. These factors are similar to those found in the patient engagement literature.^20^

The results indicate that the URP's activities cover the five objectives of the mandate, that is, the ability to provide food for thought throughout the scientific production process; the consideration of emerging concerns; the integration of issues linked to the acceptability and applicability of recommendations; the collection of data from users; and the development of knowledge transfer strategies. However, the lack of consensus on the most appropriate times to call on the panel probably deterred some agency professionals from using it. Furthermore, as the panel was targeted to meet DESA requirements, other departments did not have the opportunity to get to know it better by requesting it.

This experience also provides an opportunity to discuss the organisational arrangements that were implemented. First of all, adherence to good practices in recruiting members^16^ seems to have enabled us to select people who were able to share their experiential knowledge in a diversified and complementary way, while at the same time highlighting the need for greater sociocultural diversity. In addition, the composition of the panel meant that several people were relatives to people with a social problem than to people with the problem itself. This particularity did not come up in the interviews. However, the agency would like to continue integrating new panel members who are directly affected by a social issue.

As far as the resources allocated to run the panel were concerned, the issues at stake were above all DESA's ability to free up enough time to organise the meetings. In terms of panel participation, the adoption of an INESSS policy allowing patients, users, citizens and caregivers to be remunerated in all INESSS work has contributed to facilitate patient recruitment. This policy is based on the recommendations of the Canadian policy on patient involvement in research, which sets out guidelines for the financial reward of individuals.^21^

### 5.2 Contribution of the URP

The results indicate that, in the space of just a few years, the URP's contribution has been seen to improve DESA practices. For some scientific agency professionals and coordinators, the perception of this contribution is significant enough to request it regularly and as early as possible in the process of carrying out a mandate.

The panel has the potential to integrate the user's point of view throughout the entire process of carrying out management work, whether in terms of understanding the different dimensions of a mandate, the collection of primary data or the way in which recommendations are formulated. The fact that the panel was mainly made up of caregivers could have brought a particular dynamic to the discussions. However, at no point in the interviews conducted or in the discussion of the results did this issue come up. However, it will be interesting to see whether panels made up of people who themselves have the problems covered (and not caregivers) would produce different results.

It helps to raise awareness among INESSS members of dimensions that they may not be aware of at first glance. URP members provide a complementary perspective, imbued with a sensitivity rooted in their experience of social issues. They make agency professionals aware of the need to supplement data from the literature with primary data collected from target populations, to understand the Quebec context and put faces behind words and figures. They help to develop data collection methodologies that are adapted to target audiences, and to formulate recommendations that consider the lived experience of the people concerned.

In this way, URP members help to humanise the scientific process while maintaining its rigour. The panel members' voices give meaning to the work carried out by agency professionals, who see the impact of their work on the people concerned. In this way, the panel helps to limit the de‐emphasis of scientific work by bringing it closer to the lives of users. Thanks to the trust that has developed between panellists and scientists, the URP's terms of engagement have become more collaborative and even coconstructive over time.^22^, ^23^

In healthcare organisation, the model that most closely resembles panels is that of the Patient Advisory Councils or other types of patient committees at the strategic level. The research carried out in these contexts shows similarities with our results. In particular with regard to the operating methods of these committees, the success factors and the added value.^8^, ^9^, ^20^, ^24^, ^25^, ^26^, ^27^ They helped to identify priority issues for users and bring them to the attention of the management committee. The same results can be seen in the creation of patient committees to support research projects.^28^, ^29^, ^30^ These articles show that discussing research issues with patients enriches the way research is conceived and carried out. Researchers are led to question their research themes, to embody their research by interacting with people affected by the issues, to review their research design and to integrate patients into their research team.

Finally, it does not replace other scientific committees that exist within the INESSS, or the collection of primary data (interviews, focus groups) or consultation of key experts.

### 5.3 Challenges

The first challenge is methodological. What types of projects should be submitted to the panel? At what point in the process should they be presented? How can the URP's input be properly integrated into a report? How can we ensure that the panel is kept informed of its added value in the process? These questions lead to recommendations for good practice in the field of integrating experiential knowledge into AHTAASS work.

Another challenge concerns the tension that exists between creating a panel with a variety of expertise, or rather seeking out targeted expertise. Within the URP framework, a wide range of expertise is represented, so as to ensure a variety of profiles covering all the issues addressed. However, how can we integrate more varied social and cultural profiles? How can we involve young people in issues that concern them? Should we set up a panel dedicated to children and teenagers? Could the selection criteria have led to a recruitment bias among members of a marginalised population? Knowing the specific nature of INESSS mandate and the panel's methodological role, the URP had to possess a fairly high level of literacy. The rationale was that the participants would have to study a large quantity of documents written by researchers, government documents and so forth. In addition, URP had to interact with INESSS professionals who did not have previous experience collaborating with service users.

The third challenge is to publicise the panel's activities and see whether the model can be exported in other directions. In fact, the first years of the panel's existence were not publicised within INESSS. However, the results of this evaluation highlight a contribution that could be just as beneficial for the other two directorates (the Directorate for the Evaluation of Drugs and Technologies for Reimbursement Purposes and the Directorate for the Evaluation and Relevance of Healthcare Intervention Methods). A first possibility would be to open up the URP to subjects of interest to them. A second model would be to create a permanent users' panel in each department. And a third possibility would be to create a panel on a cross‐cutting theme such as oncology, covering drugs, intervention methods and social issues.

### 5.4 Changing roles for agencies for health technology assessment and assessment of social services

Among the contributions highlighted by URP members was a desire to democratise the knowledge produced by AHTAASS. In fact, the current positioning of these agencies leads them to respond preferentially to mandates from governments and agency professionals to improve the health and social conditions of the population.^31^ In this context, some tools can be designed to help users live better with a social or health problem, but this remains marginal. However, the more systematic consideration of users' experiential knowledge is leading to a growing awareness of the importance of working both on recommendations and tools for agency professionals, and on the development of tools for users to enable them to be proactive in dealing with their social or health problems. As a result, AHTAASS is gradually expanding its target audience.

Like INESSS, the CADTH has set up an intersectional user committee since 2019, enabling patient and caregiver experiences to be integrated into the agency's files.^32^

The National Institute for Health and Clinical Excellence, United Kingdom^33^ includes ‘lay members’ who contribute to the committee's work by passing on the thoughts of patients, people who use services, caregivers or communities. In these committees, although the majority of professional members recognised the contribution of patient involvement in helping to understand the condition, the involvement of lay members increased conflicts in decision‐making and the credibility of patients' testimonies were repeatedly questioned.^34^ In France, the Haute Autorité de Santé (HAS)^35^ includes user representatives on these professional committees, but these must be members of patient associations, to ensure ‘credibility’. However, this practice is not documented by the HAS. In the United States, the Agency for Healthcare Research and Quality^36^ does not include users in its committees, and the Institute for Clinical and Economic Review^37^ includes professional patient advocates in its independent appraisal committee, but no users. This minority representation of patients in the largest agencies coexists with the international demand for patients to be included, arguing that their presences, experiences and knowledge are important to the democratisation of evidence and the legitimacy of HTAs,^31^ and the patients' call for action, which asks that agencies and stakeholders involved in HTA to work together with patients for visionary and transformative elevation of the voice of patients in HTA worldwide.^38^

## 6 STRENGTHS AND LIMITATIONS

In terms of the study's strengths, we interviewed all the people closest to our subject. We were able to cross‐reference different sources of data, and we validated our results with the people concerned, thereby enriching our knowledge of the phenomenon under study. On the other hand, we had very little access to the agency professionals who did not use the URP to better understand the reasons behind this situation, and we interviewed only one person for the other two INESSS directorates, which did not allow us to assess certain ideas.

## 7 RECOMMENDATIONS

The results of this article also provide recommendations that can be articulated around seven dimensions: (1) the choice of members (2) moderation (3) the timing of the consultation (4) modality (5) how to integrate feedback from panel members (6) how to give them feedback on their contribution and (7) how to make a panel sustainable. In addition, cross‐cutting factors need to be considered (see Table 2).

**Table 2:** Summary of recommendations.

Dimensions	Actions
Choice of members	− Significant experience of using social services. − Diversity of backgrounds. − Ability to express oneself clearly, to listen. − Ability to step back from personal situations. − Critical thinkers. − Constructive and respectful attitude. − Good balance between people directly affected and family caregivers.
Moderation	− Coleadership between an agency professional and a user.
Timing of the consultation	− The sooner the better. − At all stages of a mandate.
Modality	− In‐person rather than remote.
Feedback integration	− Integration of comments gathered from the panel and changes made thanks to the input of the identifiable panel in the final report.
Feedback on their contribution	− Acknowledging the panel's contributions in the report. − Annual review of the panel's most important contributions. − Presentation of the panel's work to various decision‐making bodies inside the AHTAASS.
Sustainability	− Allocate financial resources to remunerate panel members. − Allocate staff time to run the panel. − Recognise the time spent consulting with panel members by scientific agency professionals as part of their task. − Renew the panel in several stages.

## 8 CONCLUSION

This article has examined the implementation of a Panel of service users in social services in an AHTAASS. It highlights how the idea came about and how it worked. It assesses the perception of its added value by panel members and by members of the INESSS and suggests avenues for improvement. It highlights the fact that such a URP enables the experiential knowledge of people affected by social problems to be integrated more systematically throughout the process of developing good practice, complementing the experiential and scientific knowledge of agency professionals. Over time, the panel's role goes beyond that of consultation, and becomes one of collaboration and even co‐construction. The panel's presence brings scientific agency professionals closer to their field of study, and reinforces the meaning given to their work. This study contributes to the development of best practices for the creation of URPs that can be implemented in contexts other than that of social services in AHTAASS.

## AUTHOR CONTRIBUTIONS

**Marie‐Pascale Pomey**: Conceptualisation; methodology; investigation; writing—original draft; writing—review and editing; supervision. **Sandra Pelaez**: Methodology; formal analysis; writing—review and editing. **Enora Le Roux**: Writing—original draft; writing—review and editing. **Oliver Demers‐Payette**: Writing—review and editing. **Marie‐Claude Sirois**: Writing—review and editing. **Louis Lochhead**: Writing—review and editing. **Isabelle Ganache**: Writing—review and editing. **Louise Normandin**: Investigation; writing—review and editing. **Audrey L'Espérance**: Writing—review and editing. **Michèle de Guise**: Writing—review and editing.

## CONFLICT OF INTEREST STATEMENT

The authors declare no conflicts of interest.

## ETHICS STATEMENT

This study received ethical approval from the Research Ethics Committee (2020‐8803, 19.350) of the of the Centre de recherche du Centre hospitalier de l'Université de Montréal.

## Supporting information

Supporting information.

Supporting information.

## Data Availability

The data that support the findings of this study are available from the corresponding author upon reasonable request.
