# Supplementary material for: How to mobilise users' experiential knowledge in the evaluation of advanced technologies and practices in Quebec? The example of the permanent users' and relatives' panel
Source: Health Expect. 2024 Jan 5;27(1):e13964. doi: 10.1111/hex.13964 (PMC10767678; doi:10.1111/hex.13964)
Supplement: Supplementary file 1 — Supporting information. [file HEX-27-e13964-s002.docx]

Annex 1 – DESA mandates having solicited panel input

| Year | Mandate | Panel contributions transcribed in the report | Link |
| --- | --- | --- | --- |
| 2021 (#1) | Characteristics and needs of targeted rehabilitation clienteles for youth with adjustment problems aged 10 or older | - Input during framing of the project - Input to define the project objectives - Input for consulting involved parties - Input on preliminary results of challenges to rehabilitation for youth with adjustment problems | <https://www.inesss.qc.ca/fileadmin/doc/INESSS/Rapports/ServicesSociaux/INESSS_Readaptation_JED_EC.pdf> |
| 2022  (#2) | Opinion on cardiopulmonary resuscitation (CPR) in the context of community-based naloxone administration for opioid overdose | - Contribution on selection criteria and diversification of users or family and friends recruited for consultations, as well as identification of potential challenges | <https://www.inesss.qc.ca/fileadmin/doc/INESSS/Rapports/Medicaments/INESSS_Avis_naloxone_RCR.pdf> |
| 2021 (#3) | Barriers and facilitators encountered by women living with a physical disability (PD), intellectual disability (ID), or autism spectrum disorder (ASD) when using perinatal services | - Input during framing of the project - Input on the state of knowledge - Input on the project orientation and on potential challenges - Proposition to conduct future work to improve practices for concerned women and their families | <https://www.inesss.qc.ca/fileadmin/doc/INESSS/Rapports/ServicesSociaux/INESSS_Perinalite_EC.pdf> |
| 2021-22  (#4) | Service pathways for youth at risk of neglect or in situations of neglect and their families (Neglect 3) | - Input on how to carry out the state of knowledge | <https://www.inesss.qc.ca/projets/projets-en-cours/fiche-projet/balises-a-une-trajectoire-de-services-pour-les-enfants-et-les-jeunes-a-risque-de-negligence-ou-en-situation-de-negligence-et-leur-famille-negligence-3.html> |
| 2020-2021 (#5) | Care pathways leading to the diagnostic assessment of neurodevelopmental disorders in children from birth until seven years old | - Continuing contribution on parental perspectives - Input on structuring of questions addressed to involved parties during consultations - Feedback on preliminary results, particularly, the importance of the support offered at each stage of the child and their family’s journey through offered services | <https://www.inesss.qc.ca/fileadmin/doc/INESSS/Rapports/ServicesSociaux/INESSS_Trajectoires_TND_EP.pdf> |
| 2020-22 (#6) | Analysis of personal and clinical characteristics associated with a favourable response to psychosocial interventions aimed at preventing and treating symptoms of common mental disorders | - Feedback on the relevance, the clinical usefulness, and the acceptance of the results for health and social service workers and professionals | <https://www.inesss.qc.ca/fileadmin/doc/INESSS/Rapports/ServicesSociaux/INESSS_TMC_EC.pdf> |
| 2020 (#7) | COVID-19 and approaches to promote compliance with precautionary and protective measures for vulnerable people | - Feedback on literature review - Raising awareness of the adaptations required for this population | <https://www.inesss.qc.ca/fileadmin/doc/INESSS/COVID-19/COVID-19_INESSS_Observance.pdf> |
| 2020 (#8) | Organization of Care and Services related to Alternate Levels of Care (ALC) | - Contribution to the debate on the problem of users waiting for ALC - Raising awareness about the experiences of people with mental health problems | <https://www.inesss.qc.ca/fileadmin/doc/INESSS/Rapports/ServicesSociaux/INESSS_NSA_EC.pdf> |
| 2020  (#9) | Autonomy in intellectually disabled youth aged 6 – 21 years old | - Rereading of the developed practice guide - Participation in the writing of the “different dimensions to consider” memory aid | <https://www.inesss.qc.ca/fileadmin/doc/INESSS/Rapports/ServicesSociaux/INESSS_DI_GP.pdf> |
| 2019 (#10) | Screening for developmental difficulties and delays in children and teenagers (0-18 years old) receiving services under the Youth Protection Act (YPA). | - Input on the acceptability and applicability of screening criteria - Spotlight on emerging issues and concerns - Raising awareness of the difficulties in accessing tests and the impact of these delays on the development of children receiving youth protection services | <https://www.inesss.qc.ca/fileadmin/doc/INESSS/Rapports/ServicesSociaux/INESSS_Depistage_retard_developpement_Avis.pdf> |
| 2019  (#11) | Reconciling the care environment and the living environment in long-term care facilities | - Consultation on the way to carry out the state of knowledge - Participation in the development of knowledge transfer tools | <https://www.inesss.qc.ca/fileadmin/doc/INESSS/Rapports/ServicesSociaux/INESSS_Conciliation_CHSLD_Etat_pratiques_2020.pdf> |
| 2019-2021 (#12) | Needs analysis grid for families followed by Integrated Perinatal and Early Childhood Services (IPECS) | - Consultation on the most appropriate methodology to use with these populations - Spotlight on the issues and concerns related to the assistance process and to the vulnerability of families supported by IPECS - Participation in the development of the family needs assessment grid for Integrated Perinatal and Early Childhood Services - Participation in writing the methods for filling in and using the grid - Participation in the creation of knowledge transfer tools - Consultation on the content of a webinar presentation to grid network members | <https://www.inesss.qc.ca/fileadmin/doc/INESSS/Rapports/ServicesSociaux/INESSS_SIPPE_GN.pdf> |
