# Supplementary material for: How to mobilise users' experiential knowledge in the evaluation of advanced technologies and practices in Quebec? The example of the permanent users' and relatives' panel
Source: Health Expect. 2024 Jan 5;27(1):e13964. doi: 10.1111/hex.13964 (PMC10767678; doi:10.1111/hex.13964)
Supplement: Supplementary file 2 — Supporting information. [file HEX-27-e13964-s001.docx]

**Appendix 2 Interview grid for Panel members**

**- Process of setting up the Panel**

How did you hear about the panel?

What motivated your interest in participating in the selection process?

What was your recruitment process like?

How were you prepared to sit on the panel, and how did this preparation meet your expectations?

**- Contribution of the Panel**

Among the projects presented during the Panel, can you give me examples of interventions where you feel you were able to shed a different light on the issue, and explain what you contributed?

Were your suggestions adopted? If so, can you give me some examples?

Were any of your suggestions not adopted? Can you give me some examples?

- **Learning from Panel members**

Can you give me examples of how you feel you have learned something from your participation in the Panel?

Have you developed any new skills since taking part in the Panel? If so, can you give me some examples?

In what ways have exchanges with other Panel members enriched your learning or not?

- **Learning from DÉSA members**

How have people at INESSS changed with regard to the input you can have on the files presented?

- **Ideas for the Panel's development over the next 3 years**.

What do you see as the positive points or areas for improvement in the Panel's operating process?

How should the Panel evolve in the short, medium and long term?

What recommendations would you offer to make the Panel even more relevant and responsive to your needs and those of the network?

**Appendix 3: Interview grid for INESSS members**

- **Process of setting up the Panel**

Where did the idea for the Panel come from?

How did you become involved in the Panel's creation?

How did you go about selecting Panel members?

How have you been prepared to lead this Panel or to support its implementation?

How were the Panel members prepared to sit on the Panel, and how did this preparation meet their expectations?

- **Contribution of the User Panel**

Among the projects presented at the Panel, can you give me examples of interventions where you feel the Panel members have shed a different light on the issue, and explain what they have contributed?

Can you give me examples of suggestions made by Panel members that were adopted? Or not? And can you tell me how this changed the way management dealt with the subject?

- **Learning by Panel members**

Can you give me examples of where panel members have developed skills during their participation in the panel?

How do members learn from each other?

On which dimensions of the Panel's mandate do you think Panel members have had the most influence? Why or why not?

- **What DÉSA members have learned**

What have you learned and/or acquired through this experience?

How have your relationships with Panel members evolved since the Panel was set up?

Do you see your work differently now that you're involved with the Panel?

- **Ideas for the Panel's development over the next 3 years**

What do you see as the positive points or areas for improvement in the Panel's operating process?

How should the Panel evolve in the short, medium and long term?

What recommendations would you offer to make the Panel even more relevant and responsive to the needs of management and the network?
